# Epstein-Barr Virus-Associated γδ T-Cell Lymphoproliferative Disorder Associated With Hypomorphic *IL2RG* Mutation

**DOI:** 10.3389/fped.2019.00015

**Published:** 2019-02-04

**Authors:** Kay Tanita, Akihiro Hoshino, Ken-Ichi Imadome, Takahiro Kamiya, Kento Inoue, Tsubasa Okano, Tzu-wen Yeh, Masakatsu Yanagimachi, Akira Shiraishi, Masataka Ishimura, Tilmann Schober, Meino Rohlfs, Masatoshi Takagi, Kohsuke Imai, Hidetoshi Takada, Shouichi Ohga, Christoph Klein, Tomohiro Morio, Hirokazu Kanegane

**Affiliations:** ^1^Department of Pediatrics and Developmental Biology, Graduate School of Medical and Dental Sciences, Tokyo Medical and Dental University, Tokyo, Japan; ^2^Department of Advanced Medicine for Virus Infections, National Center for Child Health and Development, Tokyo, Japan; ^3^Department of Pediatrics, Graduate School of Medical Sciences, Kyushu University, Fukuoka, Japan; ^4^Department of Pediatrics, Dr. von Hauner Children's Hospital, University Hospital, Ludwig-Maximilians-University Munich, Munich, Germany; ^5^Department of Community Pediatrics, Perinatal and Maternal Medicine, Graduate School of Medical and Dental Sciences, Tokyo Medical and Dental University, Tokyo, Japan; ^6^Department of Child Health, Faculty of Medicine, University of Tsukuba, Tsukuba, Japan; ^7^Department of Child Health and Development, Graduate School of Medical and Dental Sciences, Tokyo Medical and Dental University, Tokyo, Japan

**Keywords:** chronic active Epstein-Barr virus infection, γδT-cell, common γ chain, *IL2RG*, JAK/STAT pathway

## Abstract

Chronic active Epstein-Barr virus (EBV) infection (CAEBV) is an EBV-associated lymphoproliferative disease characterized by repeated or sustainable infectious mononucleosis (IM)-like symptoms. EBV is usually detected in B cells in patients who have IM or Burkitt's lymphoma and even in patients with X-linked lymphoproliferative syndrome, which is confirmed to have vulnerability to EBV infection. In contrast, EBV infects T cells (CD4^+^ T, CD8^+^ T, and γδT) or NK cells mono- or oligoclonally in CAEBV patients. It is known that the CAEBV phenotypes differ depending on which cells are infected with EBV. CAEBV is postulated to be associated with a genetic immunological abnormality, although its cause remains undefined. Here we describe a case of EBV-related γδT-cell proliferation with underlying hypomorphic *IL2RG* mutation. The immunological phenotype consisted of γδT-cell proliferation in the peripheral blood. A presence of EBV-infected B cells and γδT cells mimicked γδT-cell-type CAEBV. Although the patient had normal expression of CD132 (common γ chain), the phosphorylation of STAT was partially defective, indicating impaired activation of the downstream signal of the JAK/STAT pathway. Although the patient was not diagnosed as having CAEBV, this observation shows that CAEBV might be associated with immunological abnormality.

## Introduction

Epstein-Barr virus (EBV) infection is a very common disease that is found in >90% of all adults with a lifelong occurrence. EBV infections commonly occur asymptomatically in infants and young children, but some individuals present infectious mononucleosis (IM), which typically manifests as fever, pharyngitis with petechiae, exudative pharyngitis, lymphadenopathy, hepatosplenomegaly, and atypical lymphocytosis. EBV is usually detected in B cells from patients who have IM or Burkitt's lymphoma. Even in X-linked lymphoproliferative syndrome (XLP), which is a primary immunodeficiency disease (PID) characterized by vulnerability to EBV infection, B cells are similarly infected with EBV. On the other hand, in most patients with chronic active EBV infection (CAEBV), which is characterized by repeated or sustainable IM-like symptoms, the virus is detected in T cells (mainly in CD4^+^ T cells, and less in CD8^+^ T cells and γδT cells) or NK cells (1, 2). Hypersensitivity to mosquito bites and elevated levels of serum IgE are observed in patients with NK cell-type CAEBV (3). In contrast, in Europe and the United States, which are known to have fewer cases than Asian countries, CAEBV patients are likely to show B-cell-type infection, B-cell depletion and hypogammaglobulinemia (4). This is suggested to be due to differences in the genetic background or environmental factors, and the pathological condition may differ depending on such differences in those infected with EBV; however, the pathophysiology of this condition remains unclear.

Severe combined immunodeficiency (SCID) is a severe form of PIDs, and is defined as a combined functional disorder of both T cells and B cells, which finally results in cell-mediated and humoral immunodeficiency (5). X-linked SCID, which is a common γ chain (γc) deficiency, is the most common phenotype. As next-generation sequencing (NGS) becomes a more common diagnostic tool, the numbers of inherited immune defects might rise even further. Immunodeficiency and autoinflammatory diseases might be found to be atypical phenotypes of SCID caused by hypomorphic *IL2RG* mutation. Here, we report on a Japanese adult with recurrent respiratory infection and EBV-associated leiomyoma during childhood, who developed recurrent infection in his adolescence. The patient was diagnosed as having CAEBV-like EBV-associated γδT-cell lymphoproliferation, and was finally revealed to have *IL2RG* mutation.

## Results

### Case Presentation

The patient was a 21-years-old Japanese male with no family history suggestive of immunodeficiency. He was born to non-consanguineous Japanese parents. He had experienced recurrent respiratory infections since childhood. At the age of 6 years, he was hospitalized with EBV-associated leiomyoma in his right bronchus, and complement deficiency (C2 and C9), low T-cell count, and reduced responses to phytohemagglutinin (PHA) and concanavalin A (ConA) were also found (6). PID of unknown cause was suspected and Trimethoprim-Sulfamethoxazole (TMP-SMX) was started. He developed Yersinia enteritis at the age of 8 and pleurisy at the age of 9. After that, he did not experience severe infection for 10 years, even after discontinuing TMP-SMX at the age of 12. Chronic cough, purpura, edema, and pain of the lower limbs appeared at the age of 19. A skin biopsy was performed, which led to a diagnosis of leukocytic fragmentative vasculitis; however, immunosuppressive therapy was postponed due to his past medical history of immunodeficiency. At the age of 21, he was hospitalized with invasive *Haemophilus influenzae* infection, which had been stabilized following adequate antimicrobial therapy, and he also suffered from recurrent pneumonia caused by multiple pathogens. Extensive immunological evaluations showed dysgammaglobulinemia, with reduced IgG (608 mg/L) and IgG2 (109 mg/dL), elevated IgA (692 mg/dL), normal IgM (62 mg/dL), reduced IgE (<3 IU/mL), and reduced CH50 levels (16 U/mL) (Supplementary Table 1), along with reduced lymphocyte proliferation (PHA 6,700 cpm and ConA 4,460 cpm). Lymphocyte subpopulation analysis showed reduced T cells, a paucity of B cells, and an increase of NK cells (Table 1). In CD3^+^ T cells, a markedly increased number of γδT cells was observed, and T cells were skewed to the memory phenotype, especially central memory T cells. The kappa-deleting recombination excision circles level was low but detectable, while the T-cell receptor excision circles level was undetectable. The patient exhibited normal production of specific antibodies against varicella zoster virus (VZV), mumps, rubella, and measles.

**Table 1:** Lymphocytes profile of the patient at 21 years of age.

**Lymphocyte profile**	**% (/μL)**	**Reference value in adults**
**T CELL LINEAGES**		
T cells (CD3^+^/Lymphocytes)	58.1 (1,258)	67.8 ± 5.4 (718–2,630)
Th cells (CD4^+^/CD3^+^)	13.5 (170)	59.9 ± 9.9 (407–1,550)
Tc cells (CD8^+^/CD3^+^)	16.0 (201)	34.1 ± 8.7 (210–1,140)
CD4^+^/CD8^+^	0.84	0.8–3.0
Naïve Th cells (CD45RA^+^ CCR7^+^/CD3^+^CD4^+^)	1.9	32.3 ± 24.0
CD4^+^ T_CM_ (CD45RA^−^ CCR7^+^/CD3^+^CD4^+^)	92.2	30.3 ± 18.7
CD4^+^ T_EM_ (CD45RA^−^ CCR7^−^/CD3^+^CD4^+^)	4.13	25.3 ± 16.1
CD4^+^ T_EMRA_ (CD45RA^+^ CCR7^−^/CD3^+^CD4^+^)	1.75	12.1 ± 20.2
Naïve Tc cells (CD45RA^+^ CCR7^+^/CD3^+^CD8^+^)	13	40.1 ± 35.5
CD8^+^ T_CM_ (CD45RA^−^ CCR7^+^/CD3^+^CD8^+^)	71.9	20.8 ± 25.3
CD8^+^ T_EM_ (CD45RA^−^ CCR7^−^/CD3^+^CD8^+^)	7.2	19.7 ± 20.3
CD8^+^ T_EMRA_ (CD45RA^+^ CCR7^−^/CD3^+^CD8^+^)	7.9	19.2 ± 25.8
αβT cells (TCRαβ^+^TCRγδ^−^/CD3^+^)	28.1	89.6 ± 4.8
γδT cells (TCRαβ^−^TCRγδ^+^/CD3^+^)	71.6	5.2 ± 4.2
Double negative T cells (CD4^−^ CD8^−^/CD3^+^TCRαβ^+^)	0.83	0.77 ± 0.35
Regulatory T cells (CD25^+^IL7R^−^/CD3^+^CD4^+^)	9.16	3.11 ± 1.02
Follicular helper T cells (CD45RO^+^CXCR5^+^/CD3^+^CD4^+^)	3.06	7.02 ± 3.43
Invariant natural killer T cells (Vb11^+^Va24^+^/CD3^+^)	0.027	0.018 ± 0.012
**B CELL LINEAGES**		
B cells (CD19^+^/Lymphocytes)	2.01 (44)	12.2 ± 4.4 (110–627)
Transitional B cells (CD24^+^ CD38^+^/CD19^+^)	2.2	8.1 ± 6.5
Memory B cells (CD27^+^/CD19^+^)	45.6	18.5 ± 8.2
IgM memory B cells (CD27^+^ IgM^+^/CD19^+^)	7.47	11.2 ± 4.0
Switched memory B cells (CD27^+^ IgD^−^/CD19^+^)	36.9	13.2 ± 7.2
IgG memory B cells (CD27^+^ IgG^+^/CD19^+^)	5.43	2.4 ± 1.4
IgA memory B cells (CD27^+^ IgA^+^/CD19^+^)	11.9	3.3 ± 2.8
CD21^+^ B cells (CD20^+^/CD19^+^)	79.7	14.3 ± 5.6
Plasmablasts (CD38^+^ IgM^−^/CD19^+^)	27.6	3.2 ± 2.3
**NK CELL LINEAGE**		
NK cells (CD16^+^ CD56^+^/Lymphocytes)	33.8 (732)	13.4 ± 4.1 (82–760)

*Values above than the normal range are shown in red, and values below than the normal range are shown in blue. Th, helper T; Tc, cytotoxic T; T_CM_, central memory T; T_EM_, effector memory T; T_EMRA_, CD45RA^+^ effector memory T*.

### Virological Examination

Virus DNA quantitative tests revealed the presence of EBV in peripheral blood mononuclear cells (PBMCs) and plasma (9.0 × 10^2^ copies/μgDNA and 4.3 × 10^2^ copies/mL, respectively), and cytomegalovirus (CMV) was also detected in plasma (4.5 × 10^3^ copies/mL). EBV was detected not only in CD19^+^ B cells (2.1 × 10^4^ copies/μgDNA) but also in γδT cells (2.1 × 10^2^ copies/μgDNA). Interestingly, RT-PCR analysis demonstrated that EBV in B cells was positive for EBNA1, EBNA2, LMP1, LMP2A, and LMP2B transcripts, whereas EBV in γδT cells was positive for EBNA1, LMP1, and LMP2A, but negative for EBNA2 and LMP2B transcripts. These findings indicated that EBV in B cells showed latency III infection; however, EBV in γδT cells showed latency II. Chronological data of EBV-related antibodies were shown in Supplementary Table 2.

### Genetic Findings

**Figure 1:**
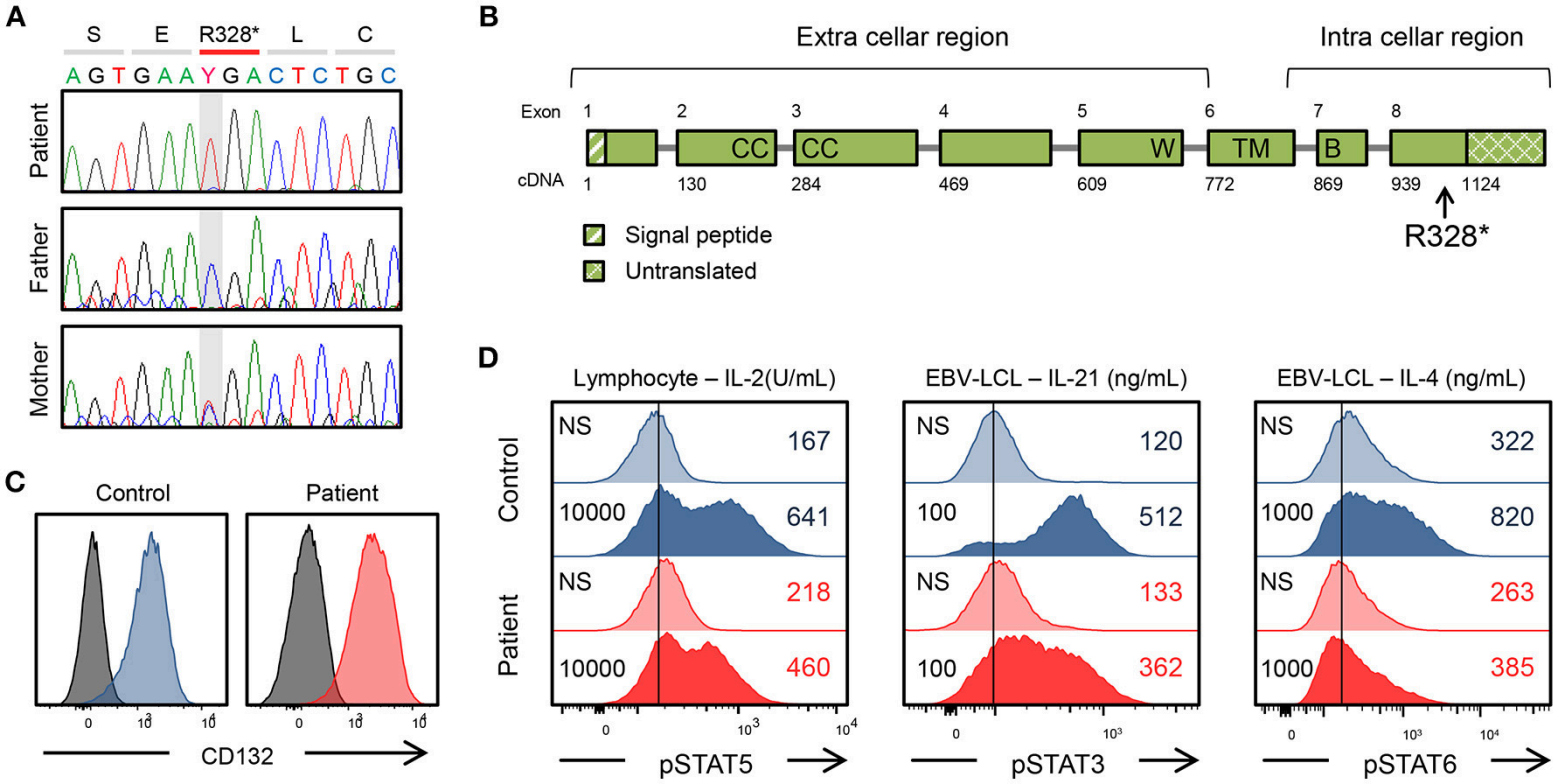
Genetic and immunological studies 1. **(A)** *IL2RG* mutation of the patient's family and *in vitro* analysis of the *IL2RG* mutant. **(B)** Gene structure of IL2RG. p.R328^*^ is located in intracellular region. W, WSEWS box; B, Box1-Box2 domain; CC, conserved cysteine; TM, transmembrane. **(C)** Surface CD132 expression in lymphocytes. **(D)** Flow cytometric analysis of pSTAT5, pSTAT3, and pSTAT6. Histogram in lymphocytes or EBV-lymphoblastoid cell line. The number on the left is the amount of cytokine. NS, non-stimulation.

Whole-exome sequencing (WES) identified a hemizygous mutation in *IL2RG* c.982C > T (p. R328^*^) in the patient. This mutation was confirmed by Sanger sequencing (Figure 1A). The mother was the heterozygous carrier of this variant. WES also revealed a homozygous mutation in C9 c.346C > T (p. R116^*^), indicating the cause of his complement deficiency.

### Immunological Findings

**Figure 2:**
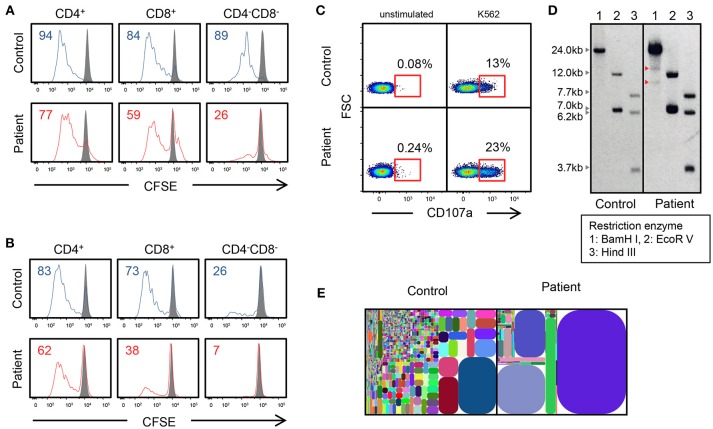
Genetic and immunological studies 2. **(A,B)** Proliferation assay of CD4^+^, CD8^+^, and CD4^−^CD8^−^ cells using CSFE after PHA and IL-2 stimulation and anti-CD3/CD28 stimulation. **(C)** CD107a expression of CD3^−^CD56^+^ NK cells, which were cultured with K562 target cells. Cells are gated on CD3^−^CD56^+^ cells. **(D)** Southern blot analysis of TCRβ chain. The patient shows extra bands (red arrowhead). **(E)** T-cell γ receptor repertoire.

The mutation was present in exon 8 of *IL2RG*, which corresponds to the intracellular domain of the γc chain (Figure 1B). Flow cytometric examination using an antibody recognizing the extracellular domain of the CD132 molecule was positive (Figure 1C). However, the phosphorylation of STAT3, STAT5, and STAT6 after cytokine stimulation was partially defective (Figure 1D). In the patient, proliferative capacity was slightly decreased in both CD4^+^ and CD8^+^ T cells, and markedly decreased in CD4^−^CD8^−^ cells which correspond to γδT cells (Figures 2A,B). The function of NK cells was normal as revealed by assessing the expression of CD107a (Figure 2C). EBV-specific CD8^+^ T cells were detectable as well as CMV-specific CD8^+^ T cells (Supplementary Figure 1). Southern blot analysis of the TCRβ chain showed extra bands in the patient, indicating that the TCRβ chain was rearranged (Figure 2D). The TCR repertoire profile showed oligoclonal expansions of γ-expressing clonotypes (Figure 2E). These findings indicated that expanded EBV-infected γδT cells might have impaired immunological function and play a pivotal role in the pathogenesis of the disease.

### Clinical Course After Diagnosis and Immunological Examination

A few months after the diagnosis, the patient presented with high fever, whole body rash with small blisters, and EBV (6.8 × 10^3^ copies/μgDNA) and VZV (1.7 × 10^4^ copies/μgDNA) viremia. The symptoms disappeared after the initiation of oral valacyclovir (VACV) for 5 days, but EBV and VZV were persistently positive in blood. Three weeks after the VACV treatment, the patient was admitted to hospital with the symptoms of high fever, cough, abdominal pain, and purpura, edema, and pain of the lower limbs. Intravenous antibiotic, acyclovir, and intravenous immunoglobulin treatment were not effective. Rituximab was also used to diminish the EBV infection in B cells, but it did not help to resolve the clinical symptoms. CMV and HHV-7 became positive along with EBV and VZV a week after admission, and the antiviral drug was switched to ganciclovir. Methylprednisolone pulse (15 mg/kg/day × 3 days) treatment was performed against hypercytokinemia [neopterin: 52 nmol/L (<5), IL-18: 3,260 pg/mL (<500), IL-6: 104 pg/mL (<5), sTNF-RI: 2,020 pg/mL (484–1,407), and sTNF-RII: 5,800 pg/mL (829–2,262)]. These treatments successfully resolved the symptoms and all four viruses became negative.

At the age of 21, the patient underwent a bone marrow transplantation from an HLA-matched unrelated donor (total nucleated cell dose 3.2 × 10^8^ cells/kg) with fludarabine at 180 mg/m^2^, melphalan at 140 mg/m^2^, etoposide at 450 mg/m^2^, and 3 Gy of total body irradiation. Graft vs. host disease (GvHD) prophylaxis with tacrolimus and short-term methotrexate were given. Although the patient achieved prompt neutrophil engraftment on day +17, acute GvHD (Grade 1: skin 2, liver 0, gut 0) developed. Additional therapy with prednisolone controlled the GvHD. Complete donor chimerism of PBMCs was demonstrated at day +21 and that of bone marrow mononuclear cells at day +29. EBV could not be detected from γδT cells, other types of T cells, B cells, NK cells, or blood plasma at day +85.

## Discussion

The protein encoded by *IL2RG* is an important signaling component of many cytokine receptors, including those of IL-1, −4, −7, −9, −15, and −21, and is thus referred to as γc (7). Mutations in *IL2RG* cause signal abnormality of these cytokines and the development of T^−^B^+^NK^−^ SCID. In the present case, although numbers of T cells and NK cells were relatively well-maintained, most of the T cells were γδ T cells lacking much of an ability to proliferate. Immunological assessment showed that phosphorylation of STAT3, STAT5, and STAT6 was partially reduced but not completely diminished. These findings suggested that this mutation (p. R328^*^) was hypomorphic.

The patient was also associated with C2 an C9 deficiency, and homozygous nonsense mutation in the *C9* was identified. C9 deficiency is the most common complement deficiency in Japan, but is very rare in western countries. The incidence of C9 deficiency was estimated to be 0.086–0.12% in Japan (8–10). Autoimmune, renal and infectious diseases were observed in some patients with C9 deficiency. The patient suffered from leukocytic fragmentative vasculitis, which might be associated with C9 deficiency.

The EBV latent infection type is classified into four types depending on the EBV genes expressed: latency I, latency II, latency III, and latency 0. Latency I is seen in Burkitt's lymphoma or nasopharyngeal carcinoma, latency II in Hodgkin lymphoma or nasal NK/T lymphoma, and latency III in opportunistic lymphoma with HIV infection and PIDs. In CAEBV, EBV infection shows latency II (11). Latency II infection in γδT cells might be compatible with CAEBV and other malignancies, and latency III infection in B cells might be compatible with PIDs.

The patient had EBV-associated leiomyoma at the age of 6 (6). EBV-positive smooth muscle tumor (SMT) is an extremely rare entity, and it is observed in patients infected with human immunodeficiency virus or undergoing immunosuppressive treatment after organ transplantation. In addition, SMT is observed in pediatric patients with PIDs including SCID (12).

**Figure 3:**
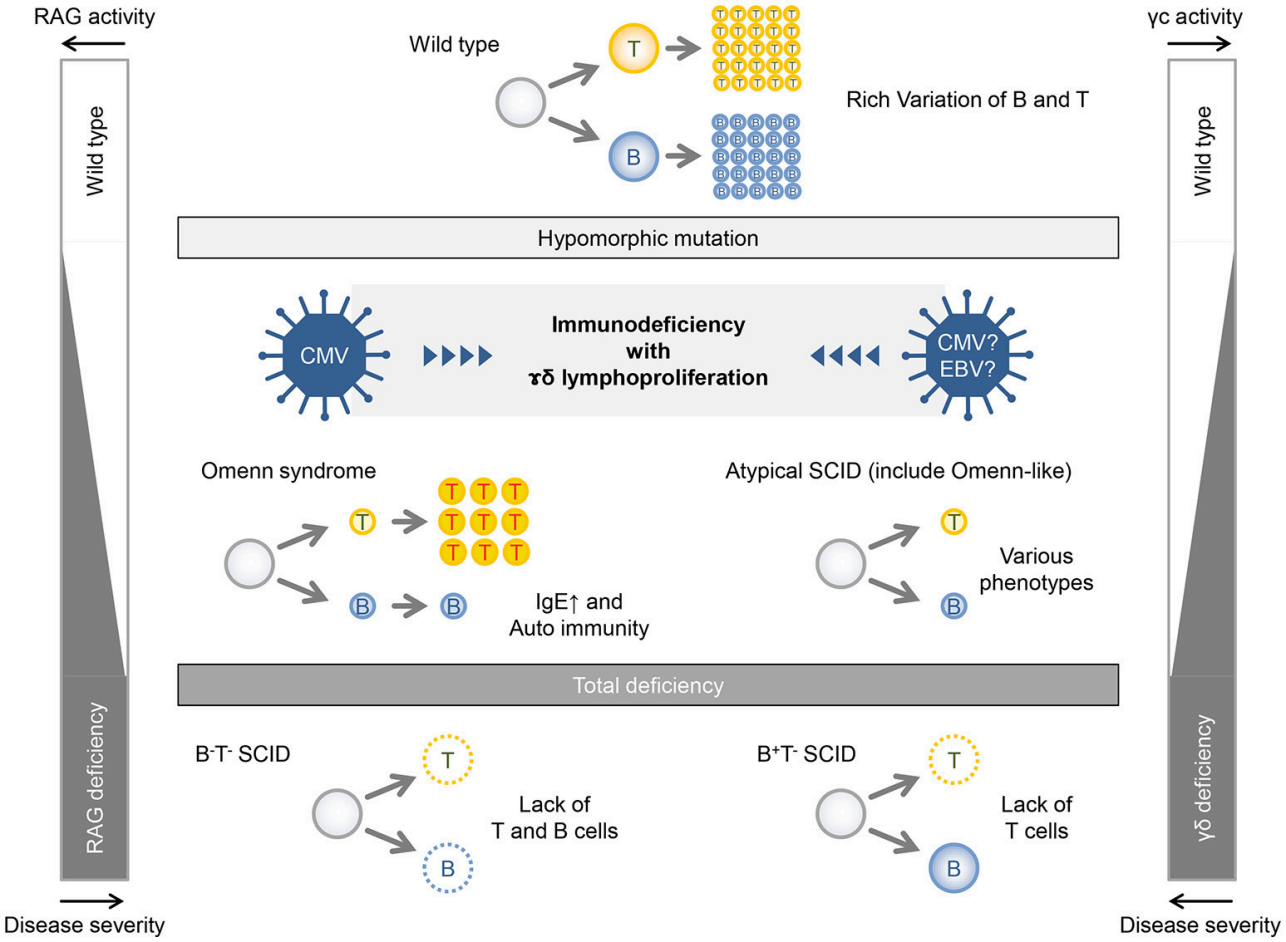
Relationship between gene activities and disease severities in patients with RAG deficiency and γc deficiency.

Recombinase activating gene (RAG)1 and RAG2 are involved in V(D)J recombination of immunoglobulin and T-cell receptor (13). Patients with complete loss-of-function mutations of *RAG1/2* genes show complete lack of T and B cells (T^−^B^−^NK^+^ SCID). On the other hand, in patients with remaining activity of RAG1/2 caused by hypomorphic mutation, B and T cells are somewhat differentiated, although they lose their diversity. This leads to the failure of immune tolerance, abnormal proliferation and activation, cytokine production biased toward Th2, and inappropriate IgE production by B-cell clones. Patients who have these conditions present with Omenn syndrome at birth (Figure 3, left panel). Patients with less hypomorphic RAG1/2 deficiency were reported to have CMV infection and γδT-cell proliferation (14, 15). In addition to the instability of immunity due to genetic abnormalities, environmental factors, such as viral infection might lead to γδT-cell proliferation. Likewise, patients with hypomorphic *IL2RG* mutation also present with an Omenn-like phenotype, while complete loss-of-function mutation in the *IL2RG* gene is linked to X-linked SCID (T^−^B^+^NK^−^ SCID) (Figure 3, right panel). Partial activity of the *IL2RG* gene makes the immunity fragile and may facilitate the infection of herpesviridae viruses, such as CMV and EBV and may feature a characteristic pathological condition of γδT-cell proliferation as well as in the case of hypomorphic RAG1/2 deficiency with CMV infection and γδT-cell proliferation. Recently, the same mutation was noted in another patient with SCID; however, the phenotypic data for that case were not reported (16). Accordingly, this is the first description of the effect of this *IL2RG* mutation.

## Concluding Remarks

The patient developed EBV-associated γδ T-cell lymphoproliferative disorder, which is virologically similar with γδ T-cell type CAEBV. The patient also presented atypical γc deficiency with hypomorphic *IL2RG* mutation. Although the diagnosis of CAEBV is made without underlying diseases including PIDs, the disease is supposed to be associated with immunological deficit. A few cases of CAEBV is associated with *PRF1* and *STXBP2* mutations (17, 18). Although the pathology of CAEBV remains unknown, the experience of this case suggests that immune abnormality is deeply involved in its onset. The accumulation of such cases should promote our understanding of the pathophysiology of CAEBV and related illness.

## Ethics Statement

This study was conducted in accordance with the Helsinki Declaration and approved by the Ethics Committee of Tokyo Medical and Dental University and written and informed parental consent was obtained for publication of this case report.

## Author Contributions

HK conceived the study. KT and HK wrote the manuscript. KT, AH, TO, and TY performed the immunological and genetic studies. K-II performed EBV studies. AH, TK, KEI, MY, AS, MI, HT, and HK were involved in the clinical care of the patient. TS and MR performed the whole exome sequencing. MT, KOI, SO, CK, and TM provided critical discussion.

### Conflict of Interest Statement

The authors declare that the research was conducted in the absence of any commercial or financial relationships that could be construed as a potential conflict of interest.

## References

[1] Cohen JI. Epstein-Barr virus infection. N Engl J Med. (2000) 343:481–92. 10.1056/NEJM200008173430707 10944566

[2] Kimura H Ito Y Kawabe S Gotoh K Takahashi Y Kojima S. EBV-associated T/NK-cell lymphoproliferative diseases in nonimmunocompromised hosts: prospective analysis of 108 cases. Blood (201) 119:673–86. 10.1182/blood-2011-10-381921 22096243

[3] Fujiwara S Kimura H Imadome K Arai A Kodama E Morio T. Current research on chronic active Epstein-Barr virus infection in Japan. Pediatr Int. (2014) 56:159–66. 10.1111/ped.12314 24528553

[4] Kimura H Cohen JI. Chronic active Epstein-Barr virus disease. Front Immunol. (2017) 8:1867. 10.3389/fimmu.2017.01867 29375552 PMC5770746

[5] Chinn IK Shearer WT. Severe combined immunodeficiency disorders. Immunol Allergy Clin North Am. (2015) 35:671–94. 10.1016/j.iac.2015.07.002 26454313

[6] Hatano M Takada H Nomura A Ohga S Ohshima K Saeki I. Epstein-Barr virus-associated bronchial leiomyoma in a boy with cellular immunodeficiency. Pediatr Pulmonol. (2006) 41:371–3. 10.1002/ppul.20375 16429426

[7] Lin JX Leonard WJ. The common cytokine receptor γ chain family of cytokines. Cold Spring Harb Perspect Biol. (2018) 10:a028449. 10.1101/cshperspect.a028449 29038115 PMC6120701

[8] Fukumori Y Yoshimura K Ohnoki S Yamaguchi H Akagaki Y Inai S. A high incidence of C9 deficiency among healthy blood donors in Osaka, Japan. Int Immunol. (1989) 1:85–9. 10.1093/intimm/1.1.85 2487678

[9] Hayama K Sugai N Tanaka S Lee S Kikuchi H Ito J. High-incidence of C9 deficiency throughout Japan: there are no significant differences in incidence among eight areas of Japan. Int Arch Allergy Appl Immunol. (1989) 90:400–4. 10.1159/000235061 2613346

[10] Kira R Ihara K Watanabe K Kanemitsu S Ahmed SU Gondo K. Molecular epidemiology of C9 deficiency heterozygotes with an Arg95Stop mutation of the C9 gene in Japan. J Hum Genet. (1999) 44:109–11. 10.1007/s100380050119 10083734

[11] Kanegane H Nomura K Miyawaki T Tosato G. Biological aspects of Epstein-Barr virus (EBV)-infected lymphocytes in chronic active EBV infection and associated malignancies. Crit Rev Oncol Hematol. (2002) 44:239–49. 10.1016/S1040-8428(02)00115-4 12467964

[12] Magg T Schober T Walz C Ley-Zaporozhan J Facchetti F Klein C. Epstein-Barr virus(+) smooth muscle tumors as manifestation of primary immunodeficiency disorders. Front Immunol. (2018) 9:368. 10.3389/fimmu.2018.00368 29535735 PMC5835094

[13] Notarangelo LD Kim MS Walter JE Lee YN. Human RAG mutations: biochemistry and clinical implications. Nat Rev Immunol. (2016) 16:234–46. 10.1038/nri.2016.28 26996199 PMC5757527

[14] Ehl S Schwarz K Enders A Duffner U Pannicke U Kühr J. A variant of SCID with specific immune responses and predominance of gamma delta T cells. J Clin Invest. (2005) 115:3140–8. 10.1172/JCI25221 16211094 PMC1242191

[15] de Villartay JP Lim A Al-Mousa H Dupont S Déchanet-Merville J Coumau-Gatbois E A novel immunodeficiency associated with hypomorphic RAG1 mutations and CMV infection. J Clin Invest. (2005) 115:3291–9. 10.1172/JCI25178 16276422 PMC1265866

[16] Luk ADW Lee PP Mao H Chan KW Chen XY Chen TX Family history of early infant death correlates with earlier age at diagnosis but not shorter time to diagnosis for severe combined immunodeficiency. Front Immunol. (2017) 8:808 10.3389/fimmu.2017.00808 28747913 PMC5506088

[17] Katano H Ali MA Patera AC Catalfamo M Jaffe ES Kimura H. Chronic active Epstein-Barr virus infection associated with mutations in perforin that impair its maturation. Blood (2004) 103:1244–52. 10.1182/blood-2003-06-2171 14576041

[18] Cohen JI Niemela JE Stoddard JL Pittaluga S Heslop H Jaffe ES. Late-onset severe chronic active EBV in a patient for five years with mutations in STXBP2 (MUNC18-2) and PRF1 (perforin 1). J Clin Immunol. (2015) 35:445–8. 10.1007/s10875-015-0168-y 25947952 PMC4504756

