# Supplementary material for: Epstein-Barr Virus-Associated γδ T-Cell Lymphoproliferative Disorder Associated With Hypomorphic IL2RG Mutation
Source: Front Pediatr. 2019 Feb 4;7:15. doi: 10.3389/fped.2019.00015 (PMC6369201; doi:10.3389/fped.2019.00015)
Supplement: Supplementary file 1 [file Data_Sheet_1.docx]

Supplementary materials for “**Epstein-Barr virus-associated γδ T-cell lymphoproliferative disorder associated with hypomorphic *IL2RG* mutation**”

**Kay Tanita, Akihiro Hoshino, Ken-Ichi Imadome, Takahiro Kamiya, Kento Inoue, Tsubasa Okano, Tzu-wen Yeh, Masakatsu Yanagimachi, Akira Shiraishi, Masataka Ishimura, Tilmann Schober, Meino Rohlfs, Masatoshi Takagi, Kohsuke Imai, Hidetoshi Takada, Shouchi Ohga, Christoph Klein, Tomohiro Morio, and Hirokazu Kanegane**

**Corresponding author**

Hirokazu Kanegane, MD, PhD

Department of Child Health and Development

Graduate School of Medical and Dental Sciences, Tokyo Medical and Dental University (TMDU)

1-5-45 Yushima, Bunkyo-ku, Tokyo 113-8519, Japan

Telephone: +81-3-5803-5244

Fax: +81-3-5803-5244

E-mail: [hkanegane.ped@tmd.ac.jp](mailto:hkanegane.ped@tmd.ac.jp)

**MATERIALS AND METHODS**

*Virological studies*

Quantitative polymerase chain reaction was performed based on the TaqMan system (Applied Biosystems, Thermo Fisher Scientific, Waltham, MA, USA). DNA was extracted from peripheral mononuclear cells (PBMCs) and plasma using an isolation kit (QIAamp DNA Mini Kit from QIAGEN for PBMC or QIAamp Min Elute Virus Spin Kit for plasma, Venlo, Netherlands). Primers and probe for real-time quantitative PCR were derived from the EBV-encoded BALF5 open reading frame: upstream primers, 5’-CGG AGG CCC TCT GGA ATG-3’; downstream primer, 5’-CCC TGT TTA TCC GAT GGA ATG-3’; fluorogenic probe, 5’-TGT ACA CGC ACG AGA AAT GCG CC-3’. Reagent kit for real-time PCR (AmpliTaq Gold DNA Polymerase with Gold Buffer & MgCl_2_ from Applied Biosystems). The identification of EBV-infected cell fractions was performed by the following method. CD19^+^, CD4^+^, CD8^+^, CD56^+^, CD14^+^, and CD3^+^ cells were serially removed from PBMCs using the IMag Cell Separation System (BD Biosciences, San Jose, CA, USA). The γδT cells were collected by the fractionation of CD3^+^ cells. DNA extraction was performed for each fraction, and quantification of EBV-DNA was performed by real-time quantitative PCR (1).

Extraction of total cellular RNA and detection of transcripts coded by EBV genes. Analysis of EBV gene expression by RT-PCR was carried out as previously described with the following primers. EBNA1: upstream primer, GAT GAG CGT TTG GGA GAG CTG ATT CTG CA; downstream primer, TCC TCG TCC ATG GTT ATC AC. EBNA2: upstream primer, AGA GGA GGT GGT AAG CGG TTC; downstream primer, TGA CGG GTT TCC AAG ACT ATC C. LMP1: upstream primer, CTC TCC TTC TCC TCC TCT TG; downstream primer, CAG GAG GGT GAT CAT CAG TA. LMP2A: upstream primer, ATG ACT CAT CTC AAC ACA TA; downstream primer, CAT GTT AGG CAA ATT GCA AA. LMP2B: upstream primer, CAG TGT AAT CTG CAC AAA GA; downstream primer, CAT GTT AGG CAA ATT GCA AA. RT-PCR primers for β-actin were purchased from Takara (Osaka, Japan). Products of RT-PCR were analyzed by agarose-gel electrophoresis and detected by staining with ethidium bromide.

*Whole-exome sequencing and Sanger sequencing*

Whole exome sequencing was performed using genomic DNA from the patient. The library was prepared using Agilent V5+UTR exome enrichment. Sequencing was performed at the Care-for-Rare Genomics Facility at the Dr. von Hauner Children’s Hospital on a NextSeq 500 platform (Illumina).

*Flow cytometric analysis*

For lymphocyte phenotyping, PBMCs were stained with fluorochrome-conjugated antibodies as previously described (2). Stained cells were analyzed using BD LSRFortessa (BD Biosciences) and the data were processed using FlowJo software (Tree Star Inc., Ashland, OR, USA). For surface CD132 (common γ chain) staining, the monoclonal antibody that recognizes the extracellular domain of CD132 [Anti-Human CD132 Biotin (TUGh4), Streptavidin-PE, and Rat IgG2b κ iso Control EB139/10H5 Biotin; eBioscience Inc., Thermo Fisher Scientific, Waltham, MA, USA] was used.

We assessed the phosphorylation of STAT3, STAT5, and STAT6 by flow cytometry. PBMCs or EBV-lymphoblastoid cell lines (LCLs) were incubated at 1 × 10^6^ cells per 100 µL in PBS. For STAT5 stimulation, PBMCs were incubated in the presence of IL-2 (100 U/mL, 1,000 U/mL) for 10 min at 37°C. For STAT3 and STAT6 cell stimulation, EBV-LCLs were also incubated in the presence of IL-21 (100 ng/mL, 10,000 ng/mL) or IL-4 (100 ng/mL, 10,000 ng/mL) for 15 min at 37°C. The stimulated cells were fixed with 4% paraformaldehyde for 10 min at 37°C, washed, and permeabilized with 90% methanol for 30 min on ice. After washing, they were stained and analyzed by LSRFortessa™. The following antibodies were used for staining: Alexa Fluor^®^ 647 Phospho-Stat3 (Tyr705) (D3A7) XP® Rabbit mAb (#4324; Cell Signaling Technology, Danvers, MA, USA), Alexa Fluor^®^ 488 Anti-Stat5 (pY694) (612598, BD Biosciences), Phospho-Stat6 (Tyr641) Antibody (#9361; Cell Signaling Technology), and Goat Anti-Rabbit IgG H&L (DyLight® 488) preadsorbed for secondary antibody for pSTAT6 (ab96899; Abcam, Cambridge, UK).

For EBV-specific CD8^+^ T cells, PBMCs were incubated with Clear Back (MBL, Nagoya, Japan) to block the Fc receptors, and stained with HLA-A*24:02 EBV Mix Tetramer-phycoerythrin (PE) (MBL) and HLA-A*24:02 CMV pp65 Tetramer-PE (MBL), followed by PE-Vio770-conjugated anti-CD8 (clone BW135/80, Miltenyi Biotec, Bergisch Gladbach, Germany) monoclonal antibodies.

*Proliferation assay*

PBMCs were labeled with 3 µM carboxyfluorescein succinimidyl ester (CFSE; Thermo Fisher Scientific) for 5 min at room temperature. CFSE-labeled cells were plated at 0.5-1.0 x 10^6^ cells/mL in triplicate (200 µL/well) in 96-well flat-bottomed plates and left unstimulated or stimulated for 4 days with anti-CD3/CD28 beads (Thermo Fisher Scientific) and PHA (Sigma Aldrich, St. Louis, MO, USA) for a T cell proliferation assay. After staining with fluorochrome-conjugated antibodies, CFSE dilution peaks of CD4^+^, CD8^+^, and CD4^-^CD8^-^ T cells were analyzed by flow cytometry as a measure of cell division. The following antibodies were used for staining: CD4-VioBlue, human and CD8-PE-Vio 770, human (Miltenyi Biotech, Bergisch Gladbach, Germany).

*CD137 expression analysis*

The capacity of T cells to respond to EBV was examined by assessing CD137 expression using flow cytometry. PBMCs were cultured with autologous EBV-LCLs at different effector-to-target ratios (1:1) for 24 h, and stained for CD8-FITC and CD137-PE (Miltenyi Biotech).

*CD107a expression analysis*

The function of NK cells was examined by assessing CD107a expression using flow cytometry. PBMCs were incubated with or without the same number of K562 as target cells in the presence of PE-anti-CD107a (BD Biosciences) for 2 h, and stained for CD3-VioGreen (Miltenyi Biotech) and CD56-PC5(Beckman Coulter, Inc., Brea, CA, USA). Cells were analyzed using flow cytometry.

*Immune repertoire sequencing*

Total RNA samples that were extracted from PBMCs were subjected to reverse-transcription PCR (RT-PCR) using a Qiagen OneStep RT-PCR kit (Qiagen) and iRepertoire^®^ human T-cell gamma receptor (HTGI-M-X-P, Human TCR gamma,V-C genes, Primers) primers (iRepertoire Inc., Huntsville, AL, USA). The detailed primer sequence information is proprietary to the company and cannot be released to the public at present. The PCR products were purified using QIAquick Gel Extraction Kit (Qiagen) and were sequenced using Illumina Mi-seq with Reagent Kits v2 (Illumina, Inc., San Diego, CA, USA). Raw data were analyzed by iRepertoire.

**REFERENCES**

1. Koizumi Y, Imadome KI, Ota Y, Minamiguchi H, Kodama Y, Watanabe D, et al. Dual Threat of Epstein-Barr Virus: an Autopsy Case Report of HIV-Positive Plasmablastic Lymphoma Complicating EBV-Associated Hemophagocytic Lymphohistiocytosis. *J Clin Immunol.*  (2018) 38: 478–483. doi: 10.1007/s10875-018-0500-4.
2. Takashima T, Okamura M, Yeh TW, Okano T, Yamashita M, Tanaka K, et al. Multicolor Flow Cytometry for the Diagnosis of Primary Immunodeficiency Diseases. *J Clin Immunol.* (2017) 37:486-495. doi: 10.1007/s10875-017-0405-7.

**Supplementary Table 1. Laboratory data of the patient at 21 years of age**

| Test | Value | Unit | Normal range |
| --- | --- | --- | --- |
| White blood cell | 5,900 | /μL | 3,500~8,700 |
| Red blood cell | 441 | x 10^4^/μL | 421~565 |
| Hemoglobin | 10.2 | g/dL | 13.5~17.1 |
| Hematocrit | 33.5 | % | 39.6~52.3 |
| Neutrophils | 57 | % | 42.0~74.0 |
| Eosinophils | 3.4 | % | 1.0~5.0 |
| Monocytes | 2.7 | % | 4.0~7.0 |
| Lymphocytes | 37 | % | 25.0~45.0 |
| Platelet | 34.5 | x 10^4^/μL | 14~33 |
| Total protein | 7.6 | g/dL | 6.7~8.3 |
| Aspartate transaminase | 32 | U/L | 13~33 |
| Alanine transaminase | 20 | U/L | 8~42 |
| Lactate dehydrogenase | 284 | U/L | 119~229 |
| Blood urea nitrogen | 12 | mg/dL | 8.0~22.0 |
| Creatinine | 0.69 | mg/dL | 0.60~1.00 |
| IgG | 608 | mg/dL | 820~1,740 |
| IgA | 692 | mg/dL | 90~400 |
| IgM | 62 | mg/dL | 31~200 |
| IgD | 18.3 | mg/dL | < 9.0 |
| IgE | < 3 | IU/mL | < 170 |
| IgG2 | 109 | mg/dL | 239~838 |
| IgG4 | 6.6 | mg/dL | 4.5~117 |
| CH50 | 16 | U/mL | 25.0~48.0 |
| C3 | 103 | mg/dL | 86~160 |
| C4 | 20 | mg/dL | 17~45 |

**Supplementary Table 2. EBV-related antibodies**

| Age | 4 years | 6 years | 7 years | 8 years | 9 years | 11 years |
| --- | --- | --- | --- | --- | --- | --- |
| VCA-IgG | 640 | 1,280 | 160 | 160 | 160 | 640 |
| VCA-IgM | < 10 | < 10 | < 10 | < 10 | < 10 | < 10 |
| VCA-IgA | NE | NE | NE | < 10 | NE | 10 |
| EADR-IgG | < 10 | 40 | 80 | 80 | 20 | 20 |
| EADR-IgA | < 10 | < 10 | NE | NE | NE | 10 |
| EBNA | 20 | 20 | 40 | 40 | 10 | 20 |

VCA, viral capsid antigen; EADR, early antigen diffuse type and related type antigen; EBNA, EBV nuclear antigen; NE, not evaluated.


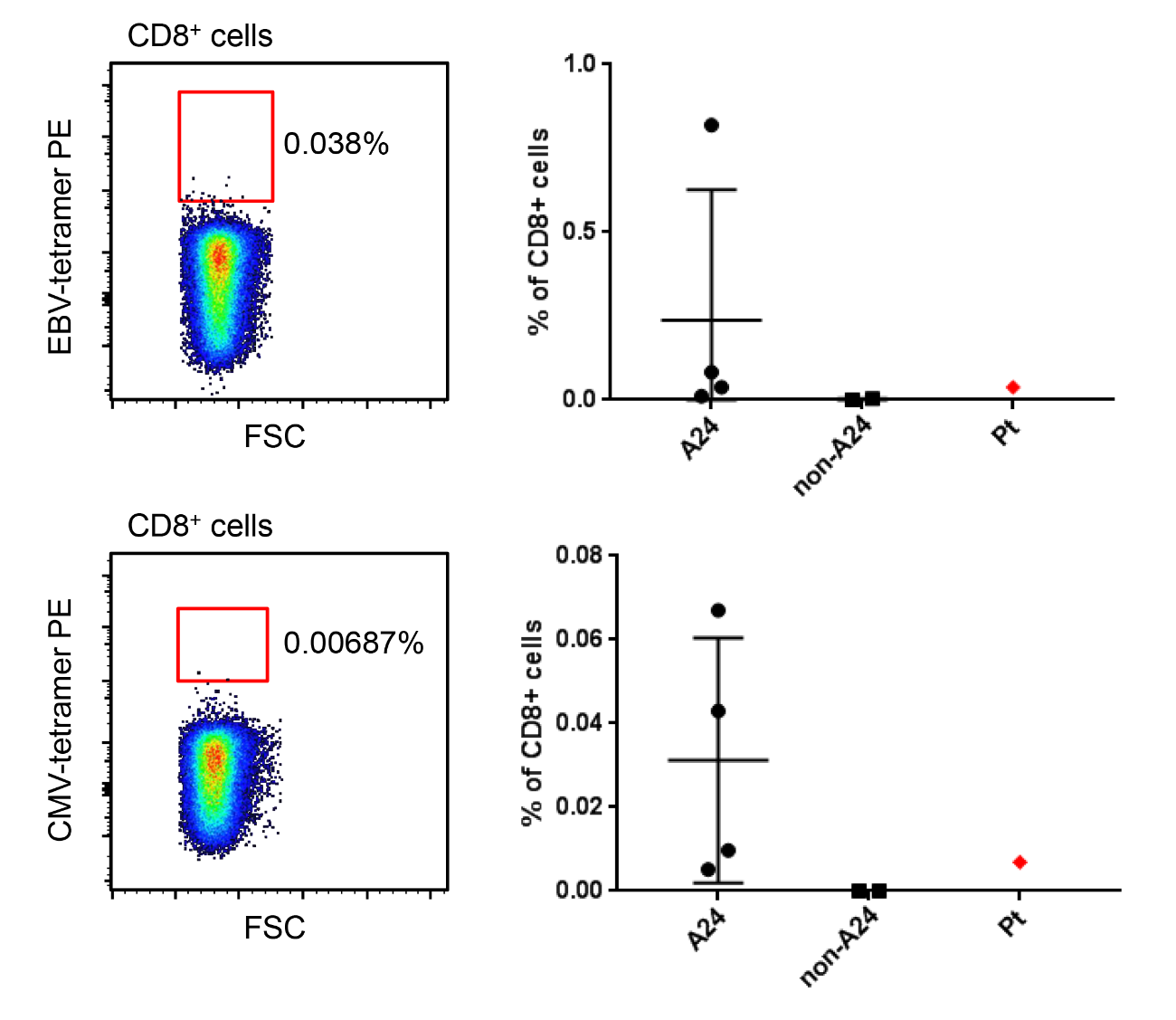


**Supplementary Figure 1. EBV-specific and CMV-specific CD8^+^ T cells.**

HLA-A*24:02 EBV mix Tetramer-positive and HLA-A*24:02 CMV pp65 Tetramer-positive cells are gated on CD8^+^ T cells. FSC, forward side scatter.
